# School-Aged Children Learn Novel Categories on the Basis of Distributional Information

**DOI:** 10.3389/fpsyg.2021.799241

**Published:** 2022-01-24

**Authors:** Iris Broedelet, Paul Boersma, Judith Rispens

**Affiliations:** Amsterdam Center for Language and Communication, University of Amsterdam, Amsterdam, Netherlands

**Keywords:** visual statistical learning, visual distributional learning, novel object categorization, statistical learning, distributional learning

## Abstract

Categorization of sensory stimuli is a vital process in understanding the world. In this paper we show that distributional learning plays a role in learning novel object categories in school-aged children. An 11-step continuum was constructed based on two novel animate objects by morphing one object into the other in 11 equal steps. Forty-nine children (7–9 years old) were subjected to one of two familiarization conditions during which they saw tokens from the continuum. The conditions differed in the position of the distributional peaks along the continuum. After familiarization it was tested how the children categorized the stimuli. Results show that, in line with our expectations, familiarization condition influenced categorization during the test phase, indicating that the frequency distribution of tokens in the input had induced novel object category formation. These results suggest that distributional learning could play an important role in categorizing sensory stimuli throughout life.

## Introduction

The world around us is incredibly complex. We need to form mental categories in order to make sense of the sensory information we perceive, which allow us to recognize and distinguish different objects, for example distinguishing a knife from a screwdriver. *Categorical perception* reflects the phenomenon that aforementioned mental categories influence how we process information: differences between objects from the same category are less important and thus more difficult to process than differences between objects from distinct categories (Harnad, 1990; Collins and Olson, 2014). Experimental studies with participants across the lifespan have demonstrated categorical perception of phenomena such as familiar objects (e.g., Newell and Bülthoff, 2002, adults), colors (e.g., Witzel and Gegenfurtner, 2016, adults), faces (Altvater-Mackensen et al., 2017, infants) and speech sounds (e.g., Liberman et al., 1957, adults; Maye et al., 2002, infants; Vandermosten et al., 2019, children). In the study of Newell and Bülthoff (2002) adult participants showed categorical perception of different familiar objects like bottles, glasses and lamps in adults. Linear continuums of three-dimensional visual stimuli were constructed, e.g., a transformation of a wine glass to a beer glass in 11 equal steps. Participants perceived these continuums as categorical rather than continuous: results from an identification task showed that there was a clear point where the object was no longer a wine glass, but a beer glass. Moreover, the experiment showed better discrimination of two tokens that surround that boundary (between-category discrimination) than of two tokens within a category (within-category discrimination).

How do humans build such mental categories? Top-down information such as linguistic labels play a role in forming object categories (Plunkett et al., 2008; Waxman and Gelman, 2009), but bottom-up learning, that is learning from low-level auditory and/or visual features without prior knowledge of the category label, is important for category formation as well. Research suggests that *statistical learning*, a learning mechanism that underlies the extraction of regularities from sensory input (Siegelman et al., 2017) contributes to bottom-up category learning, by detecting the similarities between different entities (Sloutsky, 2003). In statistical learning research, it has been shown that infants, children and adults extract regularities from the environment in the linguistic and the visual domain (e.g., Sherman et al., 2020). For example, infants are able to track the co-occurrence of shapes when exposed to complex scenes (Fiser and Aslin, 2002). In more recent work, Wu et al. (2010, 2011) showed that infants are sensitive to co-occurring visual features and that they can use this information to learn about object integrity.

A specific type of statistical learning that is important for category formation is *distributional learning*. Distributional learning is defined as learning from exposure to the relative frequency of stimuli in the environment. Maye et al. (2002, 2008) proposed the hypothesis that distributional learning underlies the formation of phonetic categories. In their experiment, 6–8-month old infants were familiarized with a speech sound continuum. Infants were subjected to one of two possible familiarization conditions. For infants in the bimodal condition, sounds from the near endpoints of the continuum were presented most frequently, whereas for infants in the unimodal condition, sounds from the middle part of the continuum were most frequent. After training, the bimodally trained infants turned out to be able to distinguish the endpoints of the continuum better from each other than the unimodally trained infants. These experiments therefore showed evidence that distributional information helps infants to acquire the sound categories that are relevant for their native language.

After the studies of Maye et al. (2002, 2008), distributional learning of phonetic categories in infants has also been found by Wanrooij et al. (2014). Moreover, evidence is reported for 8–9 year old children (Vandermosten et al., 2019) and adults (e.g., Hayes-Harb, 2007). This accumulated evidence supports the plausibility of the findings in distributional learning studies (Ter Schure et al., 2016). The study of Hayes-Harb (2007) suggests that distributional learning mechanisms also play a role when adults learn new phonetic contrasts in a second language. Vandermosten et al. (2019) found that also school-aged children can learn a new phonetic contrast based on distributional cues and that children with dyslexia seem to be less sensitive to those cues.

Infants, children and adults are thus able to build *phonetic* categories based on the distributional regularities in the input. But is this specific to phonetics or does it generalize to other cognitive domains? For example, does distributional learning also support the formation of *visual* categories? Altvater-Mackensen et al. (2017) investigated whether infants are sensitive to distributional cues when learning about new faces in an EEG study. In a design similar to Maye et al. (2002), a continuum that morphed from one female face to another was constructed and bimodal and unimodal familiarization conditions were compared. Results showed that infants in the bimodal group are better at discriminating two faces from the endpoints of the continuum compared to participants in the unimodal group, indicating that they form two categories. In another study, Junge et al. (2018) applied the research design of Maye et al. (2002) to novel object category learning. Six to eight-month-old infants were familiarized with exemplars from an 8-step continuum of two novel objects. Again, it was shown that infants that are subjected to the bimodal condition have stronger discrimination than infants that are subjected to the unimodal condition. These studies suggest that distributional learning is a domain-general learning mechanism underlying the categorization of auditory as well as visual stimuli, at least in infancy. It is yet unknown whether visual distributional learning plays a role in novel object categorization in older children as well. As the visual environment is endlessly variable and everchanging it is probable that distributional learning plays a role in learning about new object categories beyond the age of infancy.

Previous evidence thus suggests that distributional learning plays a role in the formation of categories of sounds, faces and novel objects. Distributional learning research predominantly focuses on learning in infants. Therefore it is presently unknown whether visual distributional learning plays a role in categorizing novel objects in older children. Research on the formation of phonetic categories shows that distributional learning mechanisms play a role throughout life. In the current study, we investigated whether bottom-up distributional learning contributes to categorizing novel visual stimuli in school-aged children.

Importantly, the conventional unimodal-versus-bimodal experimental design used in distributional learning studies has been criticized recently as it appears to contain a confounding factor (Wanrooij et al., 2015). Namely, when bimodal and unimodal distribution conditions are compared, not only the number of peaks differ between conditions but also the dispersion (or spreading) of the exemplars along the continuum. Specifically, in the usual distributional learning designs, the standard deviation between the stimuli in the bimodal condition is higher than that between the stimuli in the unimodal condition, which could result in better discrimination for the bimodal group. Wanrooij et al. (2015) constructed bimodal and unimodal distributions that were controlled for dispersion to test this prediction and found (when comparing the null hypothesis with four other plausible hypotheses) that it is likely that people in the bimodal condition cannot discriminate endpoint tokens better than people in the unimodal condition. The authors state that previous research on distributional learning might be unreliable because of the confounding factor of dispersion. Therefore it is important to take this factor into account.

In the current paper we adapted the design of Chládkova et al. (2020), who compared learning on two bimodal familiarization conditions (instead of comparing unimodal and bimodal conditions) in adult second language learners of Spanish. Using this design, they did not test whether participants learned two categories or one broad category, but whether they learned two different sets of two categories depending on the location of the distributional peaks in the continuum. We hypothesized that children learn that two tokens that fall within one distributional peak belong to one category, while tokens that fall into different distributional peaks belong to two different categories. As those peaks were different in the two conditions, we were able to test whether children categorize the same stimuli differently dependent on condition.

Previous evidence suggests that infants, children and adults are sensitive to auditory and visual statistical information, and on that basis we hypothesized that school-aged children are also able to learn novel object categories based on the distributional properties of the input.

## Method^1^

### Participants^2^

Fifty children (23 females, 27 males) were recruited *via* two primary schools in the Netherlands. One child was excluded from analysis because of the diagnosis of dyslexia. Their ages varied between 7;6 (years;months) and 9;9 (*M* = 8;6, *SD* = 1;1). All children were native speakers of Dutch and had been brought up monolingually. They did not have any hearing difficulties, serious visual problems, nor a diagnosis of autism spectrum disorder, AD(H)D, learning difficulties, developmental dyslexia or any other language-based disorders. Ethical approval for the experiment was obtained from the Ethical Committee of the faculty of Humanities of the University of Amsterdam. The caretakers of the children filled in an informed consent form prior to their participation. Each child was randomly assigned to one of the two familiarization conditions. As the exclusion of one child resulted in an odd number of participants, 25 children did Condition 1, while 24 did Condition 2.

### Stimuli and Design

A continuum ranging from one visual object to another in 10 equal steps was constructed using the Sqirlz 2.1 software.^3^ The endpoint stimuli (photos of two toys from Giant Microbes)^4^ were copied from Junge et al. (2018) with their permission. We constructed 9 intermediate pictures to arrive at an 11-point continuum (as opposed to the 8-point continuum that was used by Junge et al., 2018) to adapt the design of Chládkova et al. (2020).

**FIGURE 1:**
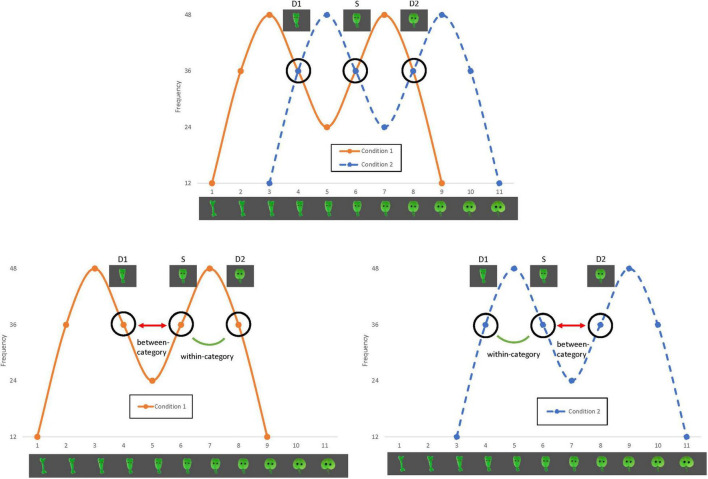
Design of the familiarization conditions of the current study. In Condition 1 (orange curve), S and D2 belong to one distributional peak, while in Condition 2 (blue curve), S and D1 belong to one distributional peak.

The familiarization phase consisted of 12 blocks in which 24 stimuli were presented (288 stimuli in total). Two conditions were developed following a between-participant design. In Condition 1 (see Figure 1, orange curve), tokens 3 and 7 were most frequent, while in Condition 2 (see Figure 1, blue curve) tokens 5 and 9 were most frequent. The frequencies of the different tokens were one, two, three, or four times per block, resulting in a total occurrence of 12, 24, 36 or 48 times after 12 blocks (see also Figure 1 for the frequencies of the different stimuli). The peaks reflected the categories in the continuum. Three of the tokens were used to test categorization after the familiarization phase, and therefore all occurred equally frequently in both familiarization conditions: token 6 [referred to as the standard (S)], token 4 [referred to as deviant 1 (D1)] and token 8 [referred to as deviant 2 (D2)]. In Condition 1, S and D2 belong to one distributional peak, while in Condition 2, S and D1 belong to one distributional peak. If the distributional properties of the input affect categorization of visual stimuli from a continuum, tokens from a distributional peak should be perceived as being more similar compared to tokens from two different peaks. The stimuli were presented in a random order, one by one against a dark grey background. Each stimulus was presented for 800 ms with an interstimulus interval of 200 ms (based on Turk-Browne et al., 2005; Arciuli and Simpson, 2011). The familiarization phase contained two randomly placed filler stimuli per block (24 in total). The filler stimuli functioned as a cover task; they moved about the screen and participants were asked to click on them as fast as they could.

**FIGURE 2:**
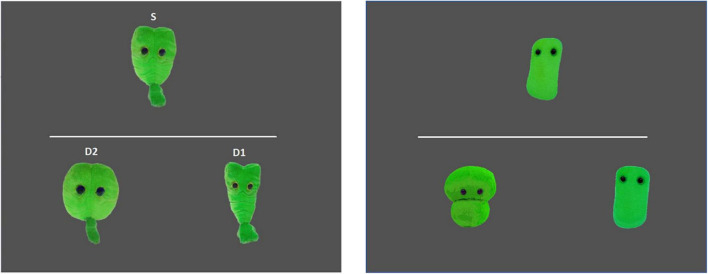
Example of a test trial and a filler/practice test trial. Participants had to choose which of the two lower pictures was a better match for the upper picture.

The test phase consisted of a practice question, eight test questions and four filler questions. We constructed AXB questions to test for categorization. In each test item, all three stimuli were presented simultaneously. S was shown in the upper part of the screen and D1 and D2 were shown below a white stripe (see Figure 2, left). All test questions were similar, but the position of D1 and D2 was counterbalanced across trials. Participants had to choose which of the two stimuli below the stripe looked more like the one above. The filler questions (Figure 2, right) were added to make the test phase less repetitive. The practice and filler questions were the same as the test questions, except that the filler stimuli from the familiarization phase were used. The test phase was the same for participants from both conditions, but the predictions were different: participants from Condition 1 were expected to have categorized S and D2 together and thus should pick stimulus D2 more often than participants who did Condition 2, who were expected to have categorized S and D1 together and thus pick D1 more often. In other words, D2 was the target answer for participants in Condition 1, while D1 was the target answer for participants in Condition 2.

### Procedure

The experiment was run in E-Prime 3.0 (Psychology Software Tools, Pittsburgh, PA). Participants sat behind a laptop wearing headphones and listened to pre-recorded child-directed instructions. They were told to watch the images on the screen carefully, and when they saw a moving image to click on it as fast as they could. They were also told there would be questions about the images, but it was not specified what type of questions. Then they were subjected to one of the two familiarization conditions. There was one short break after half of the familiarization trials. After the familiarization phase, participants did the test phase. They were instructed: “Look carefully at the image on the top of the screen. Which one of the two images below the white stripe looks more like the upper image?” The experimenter pointed toward the images and repeated: “Which one of these two images?” The test phase started with a practice question with filler stimuli. Participants used a computer mouse to answer the questions. Testing took approximately 10 min.

## Results

### Main Results

**FIGURE 3:**
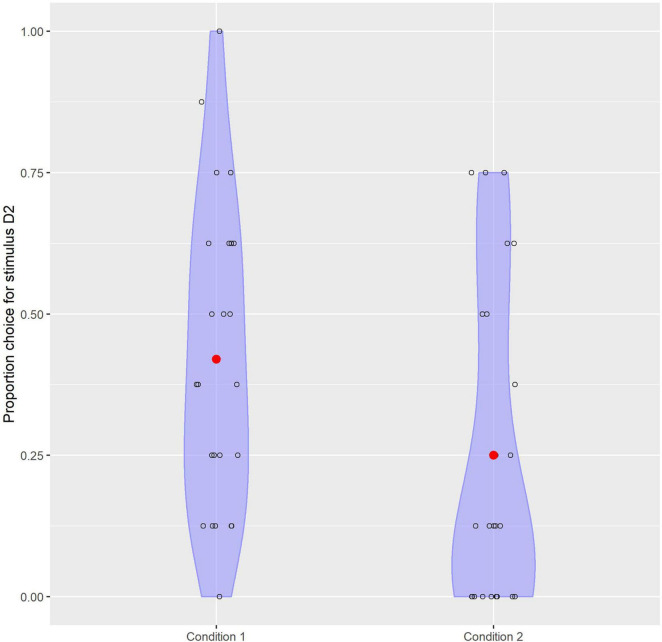
Plot depicting the choice for stimulus D2 depending on familiarization condition which shows the individual variation.

Results were analyzed in R (R Core Team, 2020). Practice and filler items were excluded for analysis. For every test item it was automatically recorded whether participants chose token D1 or D2 to look more like S. Overall, participants preferred token D1 over D2, but stimulus choice was influenced by familiarization condition. Figure 3 shows the choice for stimulus D2 per condition and includes individual variation. As the data could be conceived of as being binomially distributed, a generalized logistic linear mixed effect model (from the package *lme4*; Bates et al., 2015) was constructed to test this finding statistically. The dependent binary variable was the choice for stimulus D2 (coded as 1, D1 was coded as 0). All eight answers for all participants were taken into account. Condition was a between-participant predictor, which was coded into sum-to-zero orthogonal contrasts (Kraemer and Blasey, 2004): –1/2 for Condition 2 and +1/2 for Condition 1. A counterbalancing predictor, PositionD2, represents the position of token D2 on the screen in the test items. This is a within-participant predictor and was coded +1/2 for Left and –1/2 for Right. The model includes by-participant random intercepts, as well as by-participant random slopes for PositionD2.

**FIGURE 4:**
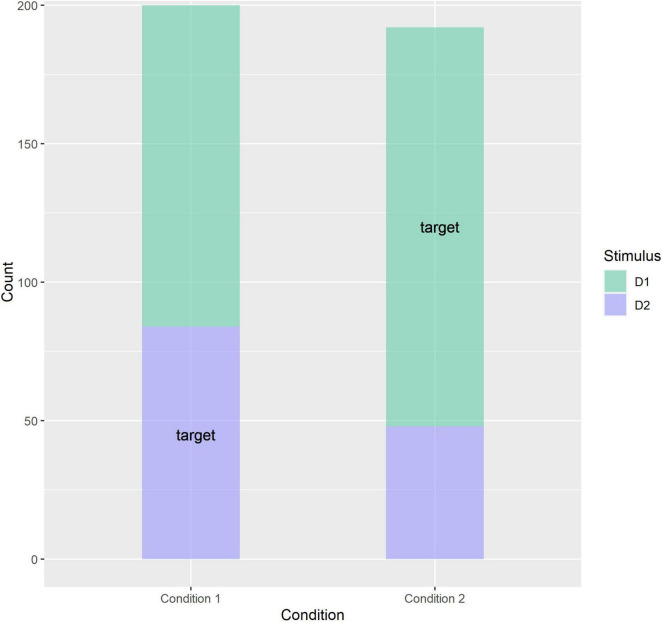
Plot depicting the choice for stimulus D1/D2 depending on familiarization condition. “Target” indicates the target answer, which is D2 for participants in Condition 1 and D1 for participants in Condition 2. Each datapoint is a single trial.

We predicted that children in Condition 1 tend to categorize tokens S and D2 together while children in Condition 2 tend to categorize tokens S and D1 together. In line with this prediction, participants in Condition 1 were 3.6 (95% CI 1.3 … 11.5) times more likely (odds ratio) to choose stimulus D2 than participants in Condition 2, and this effect of Condition was significant: *z* = 2.384, *p* = 0.017. We can conclude that familiarization condition influences the preference for combining token S with token D1 or D2, indicating that the distributional properties of the input in the familiarization phase influence categorization of the stimuli (see Table 1 and Figure 4). Participants were 2.1 (95% CI 0.97. 5.3) times more likely to choose stimulus D2 when it was positioned left on the screen as opposed to right, but this effect of PositionD2 was not significantly different from 1 (*z* = 1.781, *p* = 0.075).

**TABLE 1:** Stimulus choice depending on familiarization condition.

Condition	D1	D2	Total
1	123	85 target	208
2	144 target	48	192

*“Target” indicates the target answer, which is D2 for participants in Condition 1 and D1 for participants in Condition 2.*

### Results Follow-Up Test

The design of this experiment was based on the assumption that, ‘‘*a priori*’’ (if there is no familiarization phase), stimuli D1 and D2 are equally good candidates to categorize with stimulus S. However, the results showed an overall preference for combining stimuli S and D1. As a follow-up analysis, we constructed an online experiment to test for inherent categorization preferences. A Google Form online survey was constructed, consisting of 4 questions. In every question, stimulus S was shown and participants had to choose whether they thought stimulus D1 or D2 looked more like it. The position of the two answers was counterbalanced across questions. 32 participants filled in the survey (*M*_age_ = 30.5 years, *SD*_age_ = 1.8 years).^5^ A one-sample *t*-test revealed that the probability of choosing stimulus D1 was significantly higher than chance (50%): *t* = 6.506, *p* = 1.6⋅10^–9^ (95% CI 0.67 … 0.83). This result indicates that adults have an inherent preference for categorizing stimuli S and D1 as opposed to S and D2. This could explain the unexpected overall preference for D1 in the current study, although the choice for either D1 or D2 was still significantly influenced by familiarization condition.

## Discussion

In our study we aimed to investigate whether distributional learning contributes to categorizing visual stimuli in school-aged children. Familiarization condition significantly influenced categorization in our experiment leading us to conclude that children that are subjected to a familiarization phase in which certain tokens belong to one distributional peak are more likely to categorize those tokens together in the test phase as opposed to children that are trained in the familiarization condition in which other tokens belong to one distributional peak. This effect implies that children can form categories on the basis of distributional properties in the visual input.

Combining our results with those of Junge et al. (2018), we can conclude that distributional learning is important for visual object categorization in school-aged children as well as infants, at least in the absence of explicit labeling. Previous research has shown that infants, children and adults use statistical information to learn about the world. Categorization of sensory stimuli is an important process that seems to be supported by such statistical learning mechanisms. This has been shown in studies that investigate distributional learning of phonetic categories in infants, children and adults, as well as for infants learning face categories based on distributional properties. Junge et al. (2018) have shown that also novel object categories can be learned by infants based on distributional information. The present study shows that older children are sensitive to these distributional properties in the input when learning about new objects. Bottom-up statistical learning mechanisms may play a life-long role in understanding our environment.

Moreover, our study shows that the method of Chládkova et al. (2020), which compared to the classic unimodal-versus-bimodal design eliminates the influence of dispersion of the tokens along the continuum, also works in the visual domain. Future studies may utilize this method to investigate (visual) distributional learning in different populations.

A small shortcoming of our study, which may have reduced its sensitivity, is the bias we found in our main experiment as well as our follow-up experiment: there seems to be an inherent preference for combining certain tokens, even without familiarization. This could be due to superficial visual properties of the stimuli. For example, the standard stimulus contains horizontal stripes, which seem to be a bit more recognizable in one distractor stimulus than the other. Interestingly, biases in arising categories are also described for children learning real-life categories (e.g., Furrer and Younger, 2005). In future studies, perhaps a different continuum of visual stimuli should be constructed and tested for inherent preferences. It might be better to choose visual stimuli that are easier to control for similarity, for example 2D shapes instead of 3D pictures. Still, the training effect remains intact, revealing that the distribution of exemplars in the familiarization phase influences novel object categorization.

## Data Availability Statement

The original contributions presented in the study are publicly available. This data can be found here: doi: 10.21942/uva.c.5015285.v1.

## Ethics Statement

The studies involving human participants were reviewed and approved by the Ethics Committee of the Faculty of Humanities, University of Amsterdam. Written informed consent to participate in this study was provided by the participants’ legal guardian/next of kin.

## Author Contributions

IB, JR, and PB designed the experiments. IB recruited the participants, collected the data, and wrote the first draft of the manuscript. IB and PB analyzed the data. JR and PB provided feedback and contributed to the manuscript. All authors contributed to the article and approved the submitted version.

## Conflict of Interest

The authors declare that the research was conducted in the absence of any commercial or financial relationships that could be construed as a potential conflict of interest.

## Publisher’s Note

All claims expressed in this article are solely those of the authors and do not necessarily represent those of their affiliated organizations, or those of the publisher, the editors and the reviewers. Any product that may be evaluated in this article, or claim that may be made by its manufacturer, is not guaranteed or endorsed by the publisher.

## Footnotes

1 This experiment is also reported in the submitted article of Broedelet et al. (2021).

2 The data of a subgroup of these participants are also reported in the submitted article of Broedelet et al. (2021). In this article, typically developing children are compared to children with developmental language disorder.

3
https://sqirlz-morph.en.softonic.com/

4
www.giantmicrobes.com

5 Ideally we would have tested new participants in the same age range as the participants of the main experiment, but due to practical constraints we tested adults. Still, the result of our follow-up test shows that the bias that we found also exists without exposure to the familiarization phase.
